# Alterações Eletrocardiográficas Induzidas pela Hipotermia

**DOI:** 10.36660/abc.20200081

**Published:** 2020-06-29

**Authors:** Ana Marques, Daniel Caldeira, Alexandra Briosa, Isabel João, Hélder Pereira

**Affiliations:** 1 Hospital Garcia de Orta EPE Almada Portugal Hospital Garcia de Orta EPE,Almada - Portugal; Centro Cardiovascular Faculdade Medicina Universidade de Lisboa Lisboa Portugal Centro Cardiovascular da Universidade de Lisboa - CCUL, Laboratório de Farmacologia Clínica e Terapêutica, Faculdade Medicina, Universidade de Lisboa, Lisboa - Portugal

**Keywords:** Bradicardia, Bloqueio Atrioventricular, Eletrocardiografia/métodos, Hipotermia, Ondas Osborn

**Figura 1:**
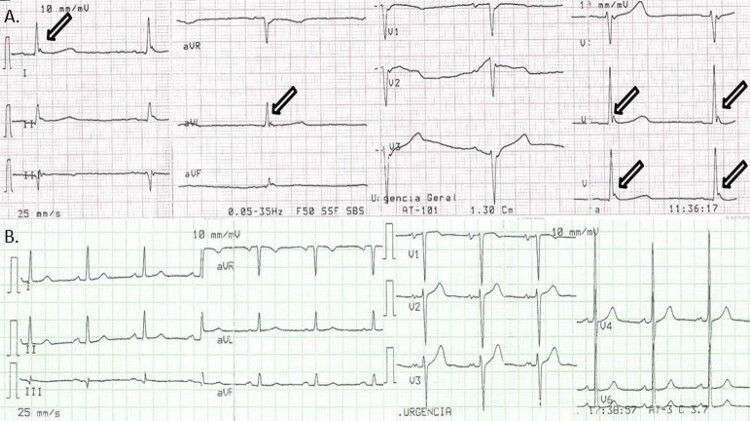
Painel A) Eletrocardiograma (ECG) mostrando alterações de ECG induzidas por hipotermia: bradicardia sinusal, bloqueio atrioventricular de 1º grau, pronlongamento do intervalo QT corrigido e ondas de Osborn no final dos complexos QRS (pontas de seta). Painel B) Após o aquecimento da paciente, o eletrocardiograma (ECG) mostra resolução das alterações eletrocardiográficas induzidas por hipotermia.

Uma paciente idosa foi levada ao pronto-socorro devido à perda de consciência. Apresentava antecedentes pessoais de hipercolesterolemia e hipertensão essencial. A paciente não era tratada com nenhum medicamento cronotrópico negativo. Na hospitalização, a pressão arterial da paciente era de 90/60 mmHg, ela apresentava bradicardia (42 bat./minuto) e hipotermia (33ºC). O eletrocardiograma (ECG) mostrou bradicardia sinusal, bloqueio atrioventricular de 1º grau, prolongamento do intervalo QT corrigido e ondas de Osborn ao final dos complexos QRS ( Figura 1 A , pontas de seta). Dessa forma, essas deflexões positivas de entalhe foram melhor observadas nas derivações precordiais laterais e desapareceram após o aquecimento da paciente a 36ºC ( Figura 1 B ). A bradicardia, o bloqueio atrioventricular e prolongamento do QT também foram resolvidos ( Figura 1 B ). Durante a internação, a tomografia computadorizada da cabeça, o Holter de 24 horas e a análise laboratorial não revelaram alterações significativas. O ecocardiograma transtorácico revelou apenas alterações degenerativas das válvulas aórtica e mitral. Este caso é ilustrativo de alterações eletrocardiográficas induzidas por hipotermia, especificamente, prolongamento dos intervalos PR, RR e QT e, principalmente, a presença de ondas de Osborn.^1 , 2^
