# Supplementary material for: Alterações Eletrocardiográficas Induzidas pela Hipotermia
Source: Arq Bras Cardiol. 2020 Jun 29;114(6):1076–7. [Article in Portuguese] doi: 10.36660/abc.20200081 (PMC8416132; doi:10.36660/abc.20200081)
Supplement: Translation

# Translation: Hypothermia-Induced Electrocardiographic Changes

**Authors:** Ana Marques, Daniel Caldeira, Alexandra Briosa, Isabel João, Hélder Pereira

**Affiliations:** 1 Portugal Hospital Garcia de Orta EPE, Almada - Portugal; Portugal Centro Cardiovascular da Universidade de Lisboa - CCUL, Laboratório de Farmacologia Clínica e Terapêutica, Faculdade Medicina, Universidade de Lisboa, Lisboa - Portugal

**Keywords:** Bradycardia, Atrioventricular Block, Electrocardiography/methods, Hypothermia, Osborn Waves

An elderly female patient was brought to the Emergency Department due to loss of consciousness. The past medical history was remarkable for hypercholesterolemia and essential hypertension. The patient was not treated with any negative chronotropic drug. At the admission, the patient’s blood pressure was 90/60 mmHg, she had bradycardia (42/minute), and hypothermia (33ºC). Electrocardiogram (ECG) showed sinus bradycardia, 1st grade atrioventricular block, long corrected QT interval, and Osborn waves at the end of QRS complexes ( Figure 1 A , arrowheads). Accordingly, these positive notching deflections were best seen in lateral precordial leads and disappeared after warming the patient to 36ºC ( Figure 1B ). The bradycardia, atrioventricular block, and QT prolongation were also resolved ( Figure 1 B ). During the inward stay, head computed tomography scan, 24-hour Holter, and laboratory analysis were unremarkable for pathological findings. Transthoracic echocardiogram only revealed degenerative aortic and mitral valve changes. This case is illustrative of hypothermia-induced electrocardiographic changes, namely prolongation of the PR, RR and QT intervals and particularly the presence of Osborn Waves.^1 , 2^

**Figure 1:** – *Panel A) Electrocardiogram (ECG) showing hypothermia-induced ECG changes: sinus bradycardia, 1st grade atrioventricular block, long corrected QT interval, and Osborn waves at the end of QRS complexes (arrowheads). Panel B) The ECG after warming the patient showing resolution of hypothermia-induced ECG changes.*
